# Commute distance and jobs-housing fit

**DOI:** 10.1007/s11116-022-10264-1

**Published:** 2022-02-18

**Authors:** Evelyn Blumenberg, Fariba Siddiq

**Affiliations:** Institute of Transportation Studies, UCLA Luskin School of Public Affairs, Los Angeles, USA

**Keywords:** Commuting, Low-wage workers, Affordable housing, Residential location

## Abstract

Anecdotal evidence suggests that the affordable housing crisis is forcing households to seek lower cost housing in the outer reaches of major metropolitan areas, helping to explain recent increases in commute distance. To test this relationship, we use spatial regression to examine the relationship between the availability of affordable housing in close proximity to jobs (jobs-housing fit) and commute distance in the Los Angeles metropolitan area. The analysis draws on 2015 Longitudinal Employer-Household Dynamics (LEHD) Origin–Destination Employment Statistics (LODES) by workplace supplemented with data from the 2013–2017 5-Year American Community Survey on affordable housing units. We find substantial variation in jobs-housing fit across Los Angeles neighborhoods. The imbalance is greatest in higher-income neighborhoods located along the coast and in Orange County, south of Los Angeles. Controlling for other determinants of commute distance, a higher ratio of jobs to affordable housing is associated with longer distance commutes. To address growing commute distances, policymakers must greatly expand and protect the supply of long-term rental housing particularly in job-rich neighborhoods.

## Introduction

Anecdotal evidence suggests that the growing affordable housing crisis in major metropolitan areas, such as Los Angeles, is forcing households to seek lower cost housing in the outer reaches of metropolitan areas, helping to explain the recent increase in commute distances (Dougherty and Burton 2017; Holder 2018; Lopez 2017; Sisson 2017; Tu 2015). Lopez (2017) writes, “Ungodly commutes, as we know, are not new to Southern California, which practically invented sprawl. People have long moved out of the city by choice, but with housing costs at historic highs, some now move by necessity.” California is one of the few states where more than 10 percent of households make hour-long or even longer daily commutes (Schuetz 2019). Workers in California coastal communities—where housing prices are highest—spend 10 percent more time commuting than the national average (Taylor 2015).

With the growing suburbanization of both jobs and households, the number of jobs within close proximity of residents has declined in most metropolitan areas (Kneebone and Holmes 2016) and commute distance has grown (Crane 2007; McGuckin and Fucci 2018). In the Los Angeles metropolitan area, from 2000 to 2012 the number of jobs near the average resident decreased by 7.4 percent, with even higher increases in commute distance in high-poverty and majority-minority neighborhoods (Kneebone and Holmes 2016). At the same time, housing prices—both home and rental—have continued to climb, even in the midst of the COVID-19 pandemic (Joint Center for Housing Studies 2020a). High housing costs may limit the ability of lower-income households to act on their preferences for living in close proximity to their jobs (Levine 1998).

Despite suggestive evidence of the relationship between housing costs and commute distance, there are relatively few studies that document this association and its magnitude. This study aims to fill this gap in the literature by examining housing availability and commute distance of workers by wages in the Los Angeles metropolitan area (Los Angeles and Orange Counties), with a focus on the commute distance of and housing available for lower-wage workers. For this analysis, we draw on data from the 2015 Longitudinal Employer-Household Dynamics (LEHD) Origin–Destination Employment Statistics (LODES) supplemented with neighborhood-level data from the 2013–2017 5-year American Community Survey (ACS).

We find substantial variation in the jobs-housing fit, the availability of affordable housing in close proximity to low- and medium-wage jobs. The imbalance is greatest in higher-income neighborhoods located along the coast and in Orange County, the county just south of Los Angeles. Moreover, controlling for other determinants of commute distance, a higher ratio of jobs to affordable housing is associated with longer distance commutes. While the availability of housing in close proximity to jobs matters for higher-wage workers, the effect is significantly muted.

Longer distance commutes can be detrimental to families as well as to the environment—adversely affecting economic mobility, activity participation, health, and air quality. The results of this study underscore the importance of available and affordable housing in enabling workers to live close to their jobs, if they so choose. However, Southern California is facing an acute shortage of affordable housing. To address increasing commute distances, policymakers must greatly expand and protect the supply of long-term rental housing particularly in job-rich neighborhoods.

## Lower-wage workers and the commute

Traditional explanations for commute distance rely on the monocentric city model and bid-rent theory (Alonso 1964; Mills 1967; Muth 1969) which posit that wealthier households tend to live in the suburbs, distant from downtown and where land prices are relatively cheap allowing for the purchase of large homes on substantial lots. Conversely, low-income households concentrate in central cities when the income elasticity of demand for land is greater than the income elasticity of travel costs. The theory predicts that higher-wage workers will travel longer distances from the suburbs to jobs in and around central cities, while lower-wage workers will live in close proximity to job centers allowing for shorter-distance commutes.

On average low-wage workers commute shorter distances than higher-wage workers (Antipova 2020; Blumenberg and King 2019; Horner and Schleith 2012; Plaut 2006; Schleith et al. 2016), potential evidence in support of this theory. However, the spatial structure of cities has changed over time with the decentralization of both households and employment (Frey 2018; Glaeser et al. 2001). Many cities have transformed from monocentric to polycentric. While some scholars have found polycentricity to be associated with shorter commutes (Gordon et al. 1986; Schleith et al. 2019; Sultana 2002; White 1990), others have found the opposite (Hamilton and Röell 1982; Schwanen et al. 2004).^1^

Although at a slower pace than among higher-income households, low-income households have suburbanized away from traditional central-city neighborhoods (Cooke and Denton 2015; Howell and Timberlake 2014; Kneebone and Garr 2010). As of 2019, more than half (52%) of the metropolitan poor lived in the suburbs, up from 46 percent in 2000 (U.S. Census Bureau, Current Population Survey 2019). At the same time, most jobs can be found outside of downtown areas (8 out of 9 jobs) and outside of employment centers (6 out of 7 jobs) (Angel and Blei 2016). In Los Angeles, in 2010 almost 56 percent of jobs were located between 10 and 35 miles from downtown (Kneebone 2013).

These changes have had ambiguous effects on commute distance particularly among low-wage workers. The suburbanization of jobs and workers has contributed to the decline in the number of jobs within a typical commute distance, a change that has been most substantial for low-income and non-white workers (Antipova 2020; Kneebone and Holmes 2016). The spatial barrier between jobs and residents has received substantial scholarly attention. The topic dates back to Kain (1968) who, in what became known as the spatial mismatch hypothesis (SMH), argued that limitations on black residential choice combined with the steady dispersal of jobs from central cities resulted in joblessness, particularly among black men.

Subsequent studies show that lower-income households who do not own automobiles tend to live in dense, transit-rich neighborhoods where they can access jobs and other destinations within a reasonable travel time (Glaeser et al. 2008; Pathak et al. 2017; Shen 2001). Although job access in central cities remains higher than in the suburbs (Shen 2001), evidence suggests that the suburbanization of jobs is slowly eroding the central-city accessibility advantage (Hu 2015). The spatial gap between central-city residents and suburban employment may contribute to longer-distance commutes, if residents are unable to adjust their residential location (Martin 2001; Stoll 2005; Weber and Sultana 2008).

However, reverse commutes from central-city neighborhoods to suburban jobs are difficult and time consuming, potentially contributing to their relatively small share of all commute trips (10%) (Davidson and Ryerson 2018). It is more likely that commute distances are growing in tandem with the increasing number of suburban poor who reside in neighborhoods with below-average numbers of jobs (Raphael and Stoll 2010) and have longer commutes than their urban counterparts (Allard and Paisner 2016; Blumenberg and King, 2019; Hu and Giuliano, 2011). Racial discrimination in both housing and employment contributes to these dynamics. For example, in their study of the spatial distribution of new jobs and residents in four metropolitan areas (Atlanta, Boston, Detroit, and Los Angeles), Stoll et al. (2000) show that residents of white suburban neighborhoods have far greater access to jobs compared to residents of black suburban neighborhoods. Moreover, research suggests that some suburban employers discriminate against black applicants, making it more likely that suburban black workers commute to central-city jobs than suburban white workers (Gottlieb and Lentnek 2001).

Finally, the lack of affordable housing may jeopardize the ability of low-wage workers to select into neighborhoods in close proximity to jobs. Numerous scholars have written about the relationship between commute distance and the jobs-housing balance—the location of employment relative to housing—asserting that more balanced cities and/or neighborhoods are linked to shorter commute distances (Cervero 1996; Peng 1997; Sultana 2002). A similar line of research centers on the relationship between the built environment (including jobs-housing balance) and “excess” commuting, defined as differences between the theoretical minimum and observed commuting distances (Hamilton 1982; Horner 2002).

In contrast, some scholars have argued that efforts to increase jobs-housing balance would have limited effects on travel, since factors other than proximity to employment (e.g. larger houses and lot sizes, improved neighborhood amenities) play a larger role in residential location choice (Giuliano 1991). Moreover, low-wage workers typically have less employment stability and, therefore, higher rates of job turnover than higher-wage workers (Lane 2000). Consequently, they are less likely than more stable workers to select neighborhoods based on the location of their current jobs and, all else equal, more likely to live farther from work (Crane 1996).

The primary benefit of jobs-housing balance may lie less in reducing overall vehicle miles of travel and much more in increasing the range of locational choices available, particularly for households with limited resources (Levine 1998). In some metropolitan areas, the lack of affordable housing may pose a significant barrier to locational choices. As we note above, housing prices—both home and rental—continue to climb (Joint Center for Housing Studies 2020a), contributing to affordable housing crises in large coastal cities (Murray and Schuetz 2018). For example, data from the 2017 5-year American Community Survey show that 56 percent of renters in the Los Angeles metropolitan area were cost burdened, spending more than 30 percent of their incomes on rent, a percentage higher than all but two of the 100 largest metropolitan areas in the country (Miami-Fort Lauderdale, FL and Riverside-San Bernardino, CA) (Joint Center for Housing Studies 2020b). In high-cost regions, the availability of affordable units relative to the location of low-wage jobs varies significantly across jurisdictions and neighborhoods within them (Benner and Karner 2016; Zonta 2020), potentially making it difficult or, in some cases, impossible for low-income households to select into neighborhoods located close to their jobs should they so choose.

## Methodology

In this analysis, therefore, we examine the relationship between the availability of affordable housing and commute distance by workplace location. We hypothesize that the availability of affordable housing—among other characteristics—is associated with shorter commute distances, particularly for low-wage workers. We test this relationship using data for the Los Angeles Metropolitan Statistical Area (MSA) which includes both Los Angeles and Orange counties.

The analysis relies on data from the 2015 Longitudinal Employer-Household Dynamics (LEHD) Origin–Destination Employment Statistics (LODES), state administrative data assembled by the Center for Economic Studies at the U.S. Census Bureau (U.S. Census Bureau 2015). The LODES data provide information on the location of workers, the location of jobs, and the location of workers relative to their jobs. Research shows that characteristics of workplace location are more strongly associated with commute behavior than characteristics of residential location (Chacon-Hurtado et al. 2019; Hu and Schneider 2017).

The LODES data report the workplace and residence locations at the census block level. The commute distance calculations are based on network distance, since variation in topography (e.g., mountains, bodies of water) and, consequently, the road network influence route choice. We identified the shortest road network path as of 2018 using the Open Source Routing Machine (OSRM), an open-source router, and the latitude and longitude coordinates for each block origin and destination pair.

We supplement these data with housing data from the 2013–2017 5-year American Community Survey. While these data are available at the block group level, we use the census tract-level data so as to minimize margins of error and, therefore, aggregated the commute distance data to this geography. The LODES data report jobs in three wage categories allowing us to calculate the network commute distance separately for lower-wage workers (with earnings $1,250 or less), medium-wage workers (with earnings $1,251/month to $3,333/month), and higher-wage workers (with earnings more than $3,333/month).

We then model median commute distance by workplace census tract and by wage group as a function of three types of characteristics—location, employment, and housing.^2^The models take the following basic form:

1 $${\text{Commute distance to workplace census tract }} = f \, (L, \, E, \, H)$$

where *L* denotes a vector of locational characteristics (distance from downtown, location in an employment center, location in an urban neighborhood, location within half mile of a rail station, distance to downtown*tract’s location in a job cluster), *E* denotes a vector of employment characteristics (the number of low- and medium-wage jobs relative to high-wage jobs, job competition denoted by the ratio of jobs and employed residents in the tract), and *H* denotes a vector of housing characteristics (median home value, vacancy rate, jobs-housing fit, and jobs-housing ratio).

To identify whether census tracts are located in an employment cluster/center, we follow the method used by Giuliano and Small (1991) and Giuliano et al. (2007). A tract is located in an employment cluster/center if it is (a) in a set of contiguous tracts (b) with a minimum 10 employees per acre and (c) together the tracts have a minimum total employment of 10,000. Commute distance into major job centers tends to be longer than commute distance into secondary job centers (Cervero and Wu 1998; Giuliano 1991). We test this by including an interaction term that relates distance to downtown and the tract’s location in a job cluster.

We identify urban and suburban tracts using the seven neighborhood types developed by Voulgaris et al. (2017). Urban neighborhoods are those that fall into three categories: old urban, mixed use, and urban residential. High activity densities in these neighborhoods are associated with less personal miles of travel and greater use of public transit. Suburban neighborhoods are those that are established suburban, patchwork, and new development.

Station locations from the Los Angeles County Metropolitan Transportation Authority (Metro) allow us to identify tracts located a half mile from a rail station. Rail-adjacent neighborhoods are varied in location and with respect to their mix of housing and jobs. For example, in their study of fixed-route transit station areas in 39 U.S. regions, Renne et al. (2016) find that 57 percent of the station areas in Los Angeles are low-density, less walkable neighborhoods. Therefore, the relationship between rail adjacency and commute distance is difficult to predict.

Historically, exclusionary land use regulations—minimum lot size requirements, single family zoning, minimum square footage requirements, and costly building codes—have been used to limit the availability of affordable housing, and have contributed to the segregation of households by income, race, and other life circumstances (Whittemore 2020). Studies show that housing costs and availability are associated with commute distance, influencing the types of workers that can select into neighborhoods in close proximity to their jobs (Cervero and Wu 1998). To test this association, our models include median home value and housing vacancy rates (the percent of vacant housing units).

In this analysis, we focus on the relationship between commute distance and jobs-housing fit, a measure of the adequacy of housing units of different prices matched to the wage characteristics of local workers. We examine commute distance among workers in the three wage groups available in the dataset: low (< $1,250/month), middle ($1,251-$3,333/month) and high (> $3,333/month).^3^ We also test the influence of jobs-housing fit relative to a general jobs-housing ratio measure (the number of all jobs relative to housing units).

To calculate jobs-housing fit, we follow the method used by Benner and Karner (2016). In addition to their low-wage jobs-housing fit metric, we also calculate a medium-wage jobs-housing fit measure to test whether housing affordability is associated with commute distance among workers in other wage categories. Recent data suggest that rising rents increasingly burden even middle-income households (Joint Center for Housing Studies 2020b).

For each of the metrics, we use a linear distance decay function to weight jobs and affordable rentals in each census tract:

2 $${\text{Low-wage jobs-housing fit for census tract}}_{i} = \frac{{\mathop \sum \nolimits_{j} {\text{Low-wage jobs}}_{j} + \mathop \sum \nolimits_{k} {\text{Low-wage jobs}}_{k} \left( {{ } - 0.4{\text{d}} + 1.2} \right)}}{{\mathop \sum \nolimits_{j} {\text{Affordable rentals}}_{j} + \mathop \sum \nolimits_{k} {\text{Affordable Rentals}}_{k} \left( {{ } - 0.4{\text{d}} + 1.2} \right)}}$$

3 $${\text{Medium-wage jobs-housing fit for census tract}}_{i} \, = \,\frac{{\mathop \sum \nolimits_{j} {\text{Medium-wage jobs}}_{j} + \mathop \sum \nolimits_{k} {\text{Medium-wage jobs}}_{k} \left( {{ } - 0.4{\text{d}} + 1.2} \right)}}{{\mathop \sum \nolimits_{j} {\text{Affordable rentals}}_{j} + \mathop \sum \nolimits_{k} {\text{Affordable Rentals}}_{k} \left( {{ } - 0.4{\text{d}} + 1.2} \right)}}$$

where *j* indicates the block groups with centroids within 0.5 miles of the population weighted centroid of census tract *i. k* indicates the block groups with centroids between 0.5 and 3.0 miles of the population weighted centroid of census tract *i,* and *d* is the straight line distance between population-weighted centroids of census tract *i* and block-group *k*.^4^

Benner and Karner (2016) used household incomes of $30,000 a year (two times the $1,250/month threshold of the low-wage job category) as the low-income threshold and $750/month (30% × $30,000/12) as the rent threshold for affordable rentals for low-income households. To enable comparisons of our findings across metropolitan areas, we use the same threshold for affordable rentals in Eq. (2) as do Benner and Karner (2016). Data on the household incomes of workers by census tract are not available. However, as a robustness check, we used data from the 2013–2017 American Community Survey Public Use Microdata Sample to calculate the median household income of renters in the Los Angeles metropolitan area with earnings less than $15,000 ($1,250 * 12); their median household incomes were $35,746 (Ruggles et al. 2021).

We set the medium-income threshold at $79,992 a year of household income (two times the $3,333/month threshold of the medium-wage job category) and the rent threshold for affordable rental for medium-income households at $1,999.8 /month (30% × $79,992/12). Rental units with rent less than $1,999.8/month and units with no cash rent are considered affordable rentals in Eq. (3). The measure is cumulative and includes units that are affordable to both middle- and low-income households. Since the 2007–09 financial crisis, renting has become increasingly common among middle-income households (United States Government Accountability Office 2020), who in tight housing markets can compete with low-income households for available units (Somerville and Mayer 2003).

As we note above, we also calculate jobs-housing ratio considering all jobs and housing units for each census tract. We apply the linear distance-decay function as follows.

4 $${\text{Jobs-housing ratio for census tract}}_{i} \, = \,\frac{{\mathop \sum \nolimits_{j} {\text{All jobs}}_{j} + \mathop \sum \nolimits_{k} {\text{All jobs}}_{k} \left( {{ } - 0.4{\text{d}} + 1.2} \right)}}{{\mathop \sum \nolimits_{j} {\text{All housing units}}_{j} + \mathop \sum \nolimits_{k} {\text{All housing units}}_{k} \left( {{ } - 0.4{\text{d}} + 1.2} \right)}}$$

where *j* indicates the block groups that have centroids within 0.5 miles of the population weighted centroid of census tract *i, k* indicates the block groups that have centroids between 0.5 and 3.0 miles of the population weighted centroid of census tract *i,* and *d* is the straight line distance between population-weighted centroids of census tract *i* and block-group *k*.

**Fig. 1:**
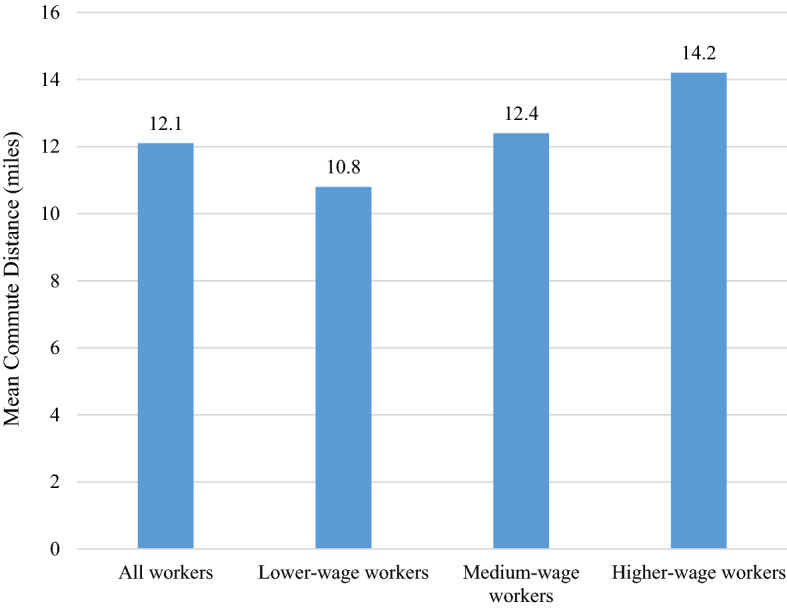
Mean Median Commute Distance by Worker Wage Group

Figure 1 shows the mean of the median commute distances across census tracts and by worker wages. As other studies find, the data reveal a positive relationship between wages and commute distance. Low-wage workers commute just under 11 miles while higher-wage workers commute more than 14 miles, 32 percent longer. Table 1 includes descriptive statistics for the dependent and independent variables in the regression models. Four of the variables—median commute distance, median home value, vacancy rate, and distance to city hall—are positively skewed and, therefore, required logarithmic transformations to approximate a normal distribution.

**Table 1:** Descriptive statistics for model variables (Workplace Census Tracts)

	Variables	Minimum	Maximum	Mean	Standard Deviation
Dependent Variables					
	Median commute distance, lower-wage workers (miles)	0.79	69.52	10.75	6.07
	Median commute distance, medium-wage workers (miles)	0.31	76.29	12.39	5.83
	Median commute distance, higher-wage workers (miles)	0.29	87.70	14.21	6.68
Independent Variables					
Housing characteristics	Median home value ($)	$10,051	$1,932,965	$547,700	$301,466
	Vacancy rate (%)	0.00	100.00	5.65	5.05
	Low-wage jobs-housing fit	0.53	173.02	6.68	7.48
	Medium-wage jobs-housing fit	0.67	14.88	1.13	0.97
	Jobs-housing ratio	0.07	7.99	1.22	0.72
Locational characteristics^†^	Distance from city hall (miles)	0.12	62.7	17.47	11.33
Employment characteristics	Number of low-wage and medium-wage jobs relative to high-wage jobs	0.08	167.00	3.73	6.45
	Competition for low-wage jobs	0.00	209.74	1.36	5.8
	Competition for medium-wage jobs	0.0038	341.1	1.71	9.95
	Competition for high-wage jobs	0.00	949.78	2.02	20.20

^†^The models also include three additional dummy variables characterizing work location (1) in an employment center, (2) in an urban neighborhood and (3) within 0.5 miles of a rail station. Eleven percent of census tracts are located in an employment center, 48 percent in an urban neighborhood, and 12 percent within 0.5 miles of a rail station

We construct three models with the following dependent variables: (1) commute distance of lower-wage workers, (2) commute distance of medium-wage workers, and (3) commute distance of higher-wage workers. We first estimate ordinary least square (OLS) regressions. For all of the models, the Moran’s I test for regression residuals suggests the presence of spatial autocorrelation and the use of spatial regression to address the issue of spatial autocorrelation.

Spatial autocorrelation can result from (1) the correlation of error terms across the spatial entities because of omitted explanatory variables, or from (2) the dependence between the dependent variable of adjacent spatial entities. The first issue can be addressed using a spatial error model and the second can be addressed by using a spatial lag model (Golgher and Voss 2016). To determine the appropriate model form, we use the Lagrange multiplier (LM) diagnostics. The statistics and significance of the robust and non-robust forms of the LM indicate that the spatial lag model was the appropriate choice.

Determining the spatial relationship among census tracts requires specification of a spatial weight matrix (Anselin 2002). We examined regression estimates based on the first-order rook contiguity, first-order queen contiguity, and distance-based spatial weights using a 3-mile distance threshold. The spatial weight matrix based on first-order queen contiguity provided the best fit. The criteria of first-order queen contiguity of shared common edge or common vertex are also reasonable definitions for assigning spatial entities as neighbors (Kawabata and Shen 2007).

The spatial lag model for all of our three models takes the following basic form:

5 $$y = \rho W_{y} + X\beta + \varepsilon$$

where, $W$ is a $n\times n$ spatial weights matrix, ${W}_{y}$ is the spatially lagged dependent variable, $\rho$ is the spatial autoregressive parameter, $X$ is an $n\times k$ matrix of observations of the independent variables, $\beta$ is a $k\times 1$ vector of regression coefficients, and $\varepsilon$ is a $n\times 1$ vector of error terms.

## Data limitations

There are a few limitations to the LODES data.^5^ First, employees who have more than one job are included in the dataset multiple times, potentially influencing the jobs-housing metrics (Horner and Schleith 2012). Further, multiple job holders likely have household incomes that are greater than the data would suggest and, if they do, would be able to afford higher-priced housing. National data suggest that the percentage of multiple job holders has increased since the Great Recession but remains relatively small, approximately 7.5 percent of all workers (Bailey and Spletzer 2021).

Second, as we describe above, the data are drawn from administrative records from the unemployment insurance (UI) program which poses two issues. While the data coverage is substantial—far more extensive than similar data from the Census Transportation Planning Products drawn from the Census and the American Community Survey—some employers are exempt from UI participation (State of California. Employment Development Department nd) and others operate in the informal sector specifically to avoid paying taxes such as UI (Nightingale and Wandner 2011). Moreover, and important for this analysis, while employers are required to report job locations, in some cases the location may not be the site at which employees work but rather the administrative headquarters of the firm. To minimize this issue, employers with multiple sites are required to submit a Multiple Worksite Report. The non-compliance rate among multi-unit employers is less than six percent (Graham et al. 2014).

Finally, the data do not indicate whether and how often workers physically travel to their places of employment, a topic of great interest during the COVID-19 pandemic. Analysis of data from the 2013–2017 American Community Survey shows that just over five percent of all workers in the Los Angeles MSA work from home. This percentage has increased over time and likely will increase further in the aftermath of the pandemic, particularly among high-skilled workers who are able to telecommute (Dey et al. 2020), potentially weakening the relevance of these data for transportation purposes.

## Jobs-housing fit

**Fig. 2:**
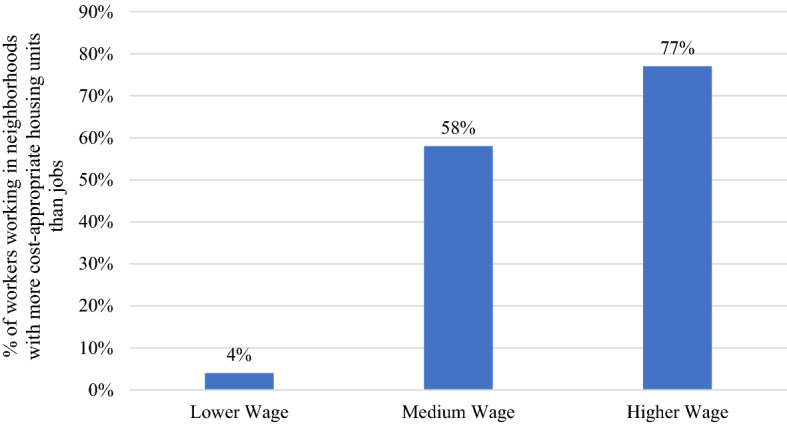
Percentage of Workers Working in Neighborhoods in which Cost-Appropriate Housing Units Exceed the Number of Workers

Figure 2 shows the percentage of workers by wage group and the presence of cost-appropriate housing. Only four percent of lower-wage workers work in neighborhoods where the number of affordable rentals exceeds the number of low-wage jobs. In comparison, 58 percent of medium wage workers and 77 percent of higher-wage workers work in neighborhoods where cost-appropriate housing exceeds the total number of jobs.

**Fig. 3:**
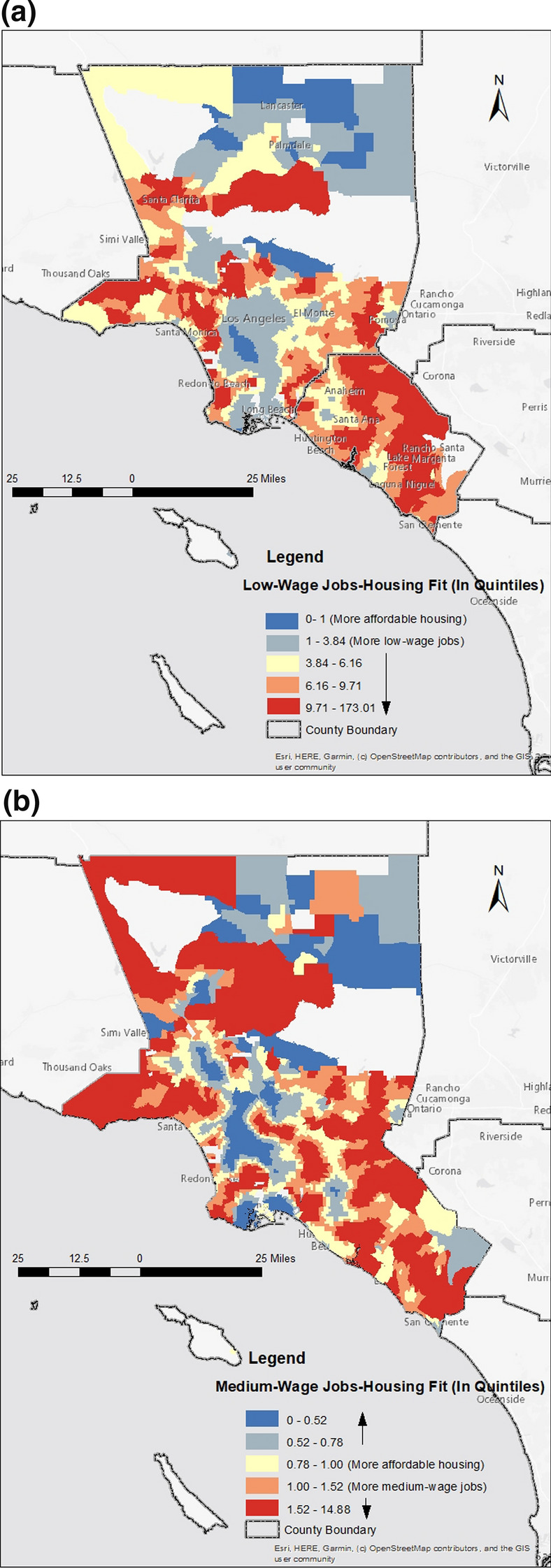

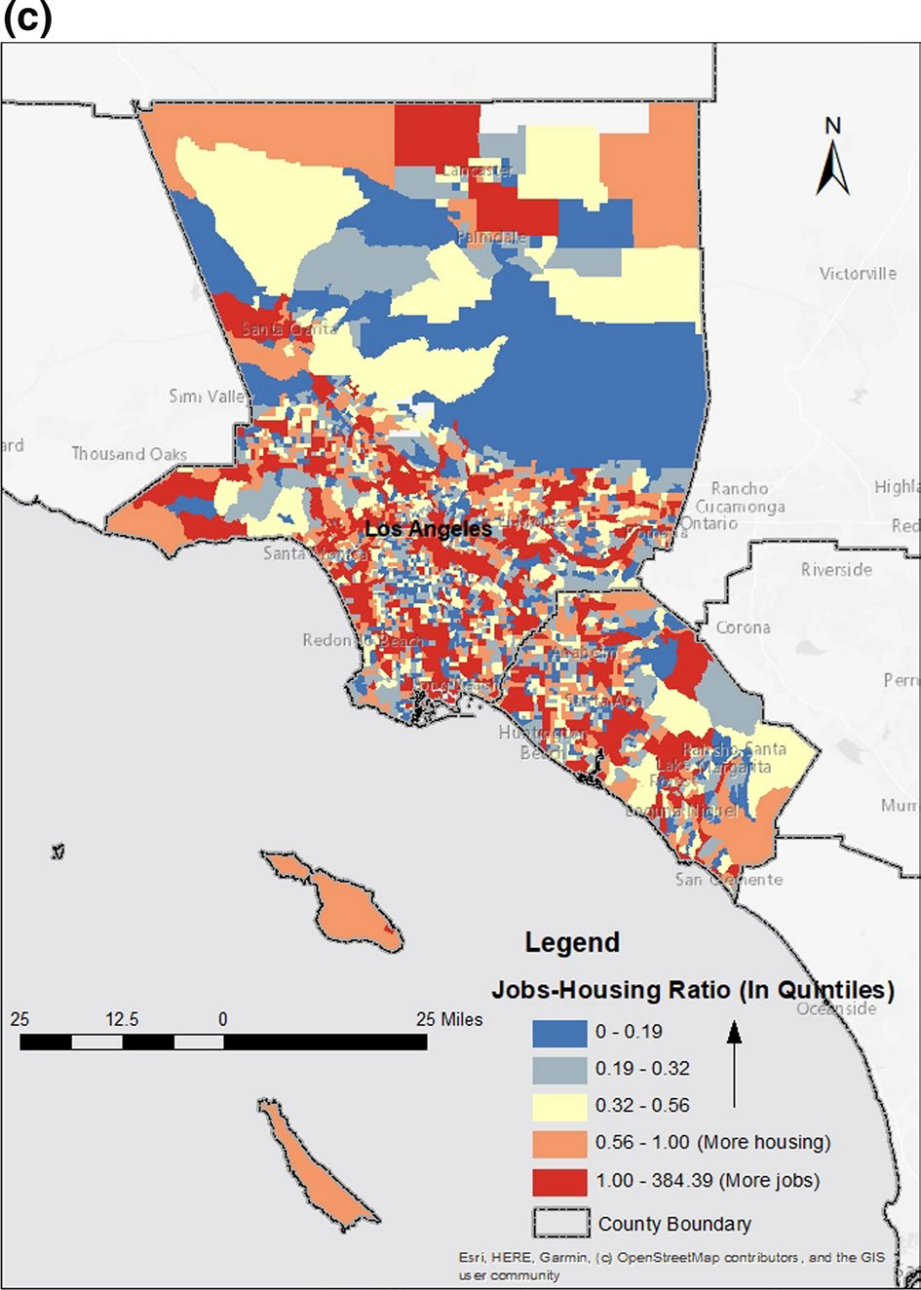
Jobs-Housing Fit, Los Angeles-Orange Metropolitan Area (Quintiles) (**a**) Low-Wage Jobs-Housing Fit (**b**) Medium-Wage Jobs-Housing Fit (**c**) Jobs-Housing Ratio

The spatial distribution of housing relative to workers varies across neighborhoods and by worker wage. Figure 3a–c include maps of our three jobs-housing fit measures by quintile. Areas in blue have more housing relative to jobs and areas in red have more jobs relative to affordable housing. For lower-wage workers, as we might expect, the supply of affordable units relative to low-wage jobs tends to cluster in the central part of the metropolitan area in close proximity to downtown Los Angeles and in the north–south corridor between downtown Los Angeles and the City of Long Beach, including the large port complex to the south. There is also a concentration of affordable rentals relative to low-wage jobs in Lancaster and Palmdale, suburban cities located 70 miles north of downtown Los Angeles. Neighborhoods along the coast and toward the south in Orange County, more affluent parts of the region, tend to have less affordable housing relative to jobs.

In addition to these areas, Fig. 3b shows that medium-wage workers can more easily locate housing in neighborhoods in the San Fernando Valley (immediately north of the LA basin) and near Anaheim in Orange County. Finally, Fig. 3c depicts the relationship between all housing and all jobs. Although the pattern is less obvious than in the previous two maps, a comparison across all three maps shows that for higher-wage workers there are additional neighborhoods with a surplus of housing relative to jobs. Of course, some neighborhoods—regardless of worker wages—have fewer housing units than jobs. These trends are a product of historical zoning regulations aimed at separating conflicting land use and neighborhood anti-development efforts often motivated by preservation and environmental interests (Fischel 2004; Glaeser 2017).

## Affordable housing and commute distance

Table 2 presents the results of the OLS and spatial lag models. The Akaike information criteria (AIC) for the spatial lag models for all wage categories are lower than the AIC values for the corresponding OLS models. This suggests that the spatial lag models have better model fits than the OLS models. The magnitudes of effects of the independent variables on commute patterns in all the models are smaller for the spatial lag model than the OLS model. The R-squared values of the OLS models suggest that the variables explain the commute distance of lower-wage and medium-wage workers better than that of higher-wage workers, likely because higher-income households have the means to act on a more diverse set of preferences (e.g., living by the beach, working from home, etc.).

**Table 2:** Estimation Results for OLS and Spatial Lag Models

Independent Variables	Lower− wage workers		Medium− wage workers		Higher− wage workers	
	OLS	Spatial Lag	OLS	Spatial Lag	OLS	Spatial Lag
Intercept	** − 0.971** **(0.136)**	**− 0.585** **(0.129)**	**− 0.372 (0.104)**	**− 0.243** **(0.101)**	**1.420 (0.114)**	**1.171** **(0.117)**
***Housing***						
Log (Median home value)	**0.168** **(0.019)**	**0.096** **(0.018)**	**0.086 (0.015)**	**0.056** **(0.015)**	**− 0.139 (0.017)**	**− 0.119** **(0.017)**
Log (Vacancy rate)	**0.0304** **(0.012)**	**0.026** **(0.011)**	**0.0303 (0.009)**	**0.021** **(0.009)**	**0.049 (0.011)**	**0.045** **(0.010)**
Low-wage jobs-housing fit	**0.005 (0.001)**	**0.003** **(0.001)**				
Medium-wage jobs-housing fit			**0.0319** **(0.004)**	**0.022** **(0.004)**		
Jobs-housing ratio					**0.016 (0.005)**	**0.012** **(0.005)**
***Locational Characteristics***						
Employment center dummy	**0.399 (0.154)**	0.237 (0.144)	**0.387** **(0.122)**	**0.263** **(0.117)**	*0.260 (0.137)*	0.201 (0.135)
Log (Distance from city hall)	**0.192 (0.014)**	**0.118** **(0.014)**	**0.182 (0.011)**	**0.125** **(0.012)**	**0.091 (0.012)**	**0.074** **(0.012)**
Employment center* log (Distance from city hall)	*− 0.064 (0.033)*	− 0.034 (0.031)	**− 0.074 (0.026)**	*− 0.049* *(0.025)*	*− 0.051 (0.029)*	− 0.038 (0.029)
Urban neighborhood	**− 0.0276** **(0.008)**	**− 0.023** **(0.008)**	− 0.009 (0.006)	− 0.006 (0.006)	**− 0.016 (0.007)**	*− 0.0126* *(0.007)*
Within 0.5 miles of rail station	**0.0507** **(0.013)**	**0.035** **(0.012)**	0.021 (0.010)	0.014 (0.010)	0.010 (0.011)	0.009 (0.011)
***Employment Characteristics***						
Ratio of low-wage and medium-wage jobs relative to high-wage jobs	**− 0.005** **(0.001)**	**− 0.004** **(0.001)**	**− 0.003** **(0.000)**	**− 0.002** **(0.000**)	**− 0.002 (0.001)**	**− 0.002** **(0.001)**
Competition for low-wage jobs	**0.011 (0.002)**	**0.011** **(0.002)**				
Competition for medium-wage jobs			**0.005 (0.001)**	**0.005** **(0.001)**		
Competition for high-wage jobs					**0.004 (0.001)**	**0.004** **(0.001)**
**R**^**2**^	0.28		0.24		0.07	
**Rho:**		0.413		0.317		0.197
**LR test value:**		278.02		145.12		46.67
**p-value:**		0.000		0.000		0.000
**Log likelihood**		797.539		1361.0		999.007
**AIC**	− 1293.1	− 1569.1	− 2552.9	− 2696	− 1927.3	− 1972

Values shown in bold are significant at p < 0.05. Values shown in italics are significant at p < 0.10. Standard errors are in parentheses

In general, the control variables perform as we predicted. All else equal, workers who worked in census tracts that are in an employment center had longer commutes, a finding consistent with earlier research (Giuliano and Small 1991). Distance to downtown is positively associated with commute distance; however, the interaction term between distance to downtown and employment center is negative. In other words, longer commutes to jobs located in downtown are partially offset in job centers located in other parts of the region.

Controlling for other variables, the association between the urban neighborhood dummy variable and commute distance is negative. As we might expect, workers who worked in urban census tracts had shorter commutes than workers in other locations. Urban neighborhoods are denser and have more diverse land use than their suburban counterparts and, therefore, jobs and residences are more likely to be located in close proximity (Cheshire et al. 2018; Schleith et al. 2016).

Commute distance was longer for workers in jobs located within half mile of a rail station, although this variable is not significant in the medium and higher-wage worker models. Rail transit may improve regional connectivity and, therefore, contribute to the growing separation of jobs and housing (Gao et al. 2019). With respect to lower-wage workers, gentrification around rail station areas may limit their ability to remain or to move into these neighborhoods, although the evidence of this behavior is mixed and may vary across station areas (Baker and Lee 2019; Boarnet et al. 2018; Dominie 2012; Kahn 2007).

The association between commute distance and competition for wage-appropriate jobs is positive indicating that census tracts with more jobs relative to employed residents likely attract workers from more distant neighborhoods. On average, an increase in the proportion of low- and medium-wage jobs compared to high-wage jobs in a tract was associated with shorter commutes. Since lower-income workers commute shorter distances than higher-income workers (Blumenberg and King 2019), workers in census tracts with higher numbers of low-wage jobs tend to have shorter average commute distances. The effect of this variable also suggests a role for skills match in residential location choice.

All of the housing variables are statistically significant in the models for all three wage categories. The distance traveled by lower- and medium-wage workers to workplace locations is positively associated with the median home value at the workplace census tract. The lack of reasonably priced housing options near employment locations likely forces lower- and medium-wage workers to live far from job locations and, consequently, leads to longer commutes. For higher-wage workers, however, commute distance is inversely related with median home value. Higher-wage workers are less likely to face cost barriers in the housing market. Commute distance and vacancy rates are positively related for all wage categories. Restrictive land use regulations limit the supply of affordable housing (Cervero 1989) and, at the same time, are associated with higher vacancy rates and longer distance commutes (Cheshire et al. 2018).

**Fig. 4:**
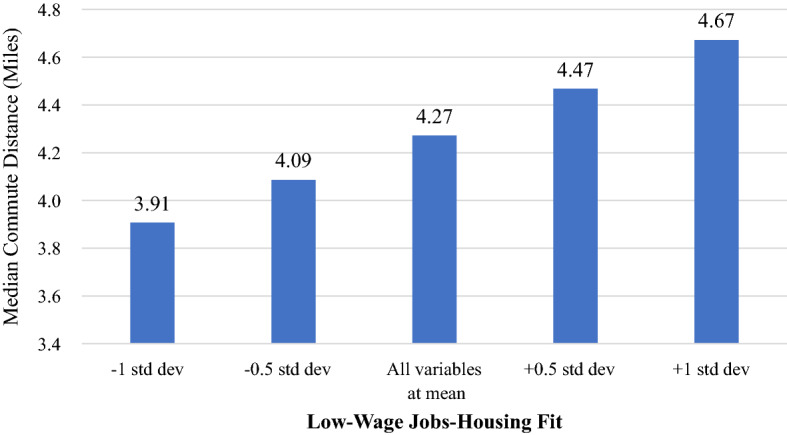
Commute Distance of Lower-Wage Workers Relative to Change in Low-Wage Jobs-Housing Fit Metric

We are particularly interested in the match between the housing affordability and jobs by wage group. The jobs-housing fit metric for lower- and medium-wage workers is positively associated with commute distance. In addition, the availability of all jobs relative to all housing is positive and statistically significant in the higher-wage workers model. However, compared to the jobs-housing fit variables in the previous two models, the availability of all jobs relative to all housing contributes far less to explaining commute distance relative to the other variables in the model.^6^ Figure 4 shows the effect of a one standard deviation change in the jobs-housing fit metric below and above the mean on the commute distance of lower-wage workers, holding all other variables at the mean.^7^ A large shift in the jobs-housing fit metric results in 19.4 percent change in the commute distance of lower-wage workers.

Finally, we tested the relationship between commute distance for lower- and medium-wage workers and the jobs-housing ratio (without accounting for fit). The findings from these models are in Table 2 in the Appendix. The jobs-housing variable is statistically significant in both models; however, the models with jobs-housing fit yield higher R-squared values, underscoring the importance of the match between workers by wage and the local availability of affordable housing.

## Conclusion

Many workers with longer distance commutes are compensated for their travel with higher wages, lower-cost housing, or other neighborhood amenities (e.g. better schools, more open space, etc.). However, the models presented here suggest that holding other workplace characteristics constant, the character and affordability of housing may not adequately meet worker demand—particularly demand from lower- and medium-wage workers. Neighborhoods with more affordable housing relative to jobs are associated with shorter commutes, suggesting that the availability of affordable housing allows, at least some households, to self-select into neighborhoods in close proximity to their jobs.

Additional data and research are needed to further substantiate and build on these findings. A principal weakness with the LODES data is the wage categories. The three categories are defined nationally and, therefore, may not adequately capture three distinct segments of the labor market in some, particularly high-cost, metropolitan areas. The Census Bureau also does not adjust the wage categories for inflation, limiting the ability of researchers to track changes over time in the commute distance of workers by wages. Finally, the origin–destination data include only limited information on worker characteristics (age group, wage category, and major employment sector) further narrowing the usefulness of the data. These limitations combined with those discussed previously suggest needed improvements in the organization and availability of these data. They also underscore the importance of supplementing research based on aggregate data with studies that draw from other data sources. The gold standard for this line of research would be longitudinal microdata that follow workers and their residential and employment choices over time.

The findings from this study, however, suggest that the lack of affordable housing contributes to longer-distance commutes, potentially with adverse consequences particularly for lower-wage workers. Evidence shows that lower-skilled workers benefit from living in high-employment-growth areas, suggesting that longer-commutes may negatively affect their economic mobility (Acolin and Wachter 2017). Workplaces located further from home may make it difficult for parents to juggle household and work responsibilities (Hanson 2010), potentially resulting in higher rates of job turnover. Longer distance commutes can burden households by taking up hours of the day that could be devoted to other productive uses such as additional paid work or spending time with family and friends (Morris et al. 2020). Finally, long distance commutes, particularly those in automobiles, can have negative health impacts such as death and injury from crashes and cardio-respiratory illnesses caused by air pollution (Douglas et al. 2011).

The findings from this study underscore the importance of policy efforts to protect and expand the supply of long-term rental housing particularly in job-rich neighborhoods located in large and expensive coastal cities. How best to do this is currently the subject of substantial debate (Manville et al. 2020; Rodríguez-Pose and Storper 2020) and beyond the scope of this study. However, broad strategies include the creation and preservation of dedicated affordable housing units, zoning changes that allow for additional housing development, the relaxation of housing standards to facilitate higher-density development, increased government housing subsidies, and tenant protections.

## Appendix 1

See Tables 3 and 4

**Table 3:** Standardized Coefficients for OLS Models

	Lower-wage workers	Medium-wage workers	Higher-wage workers
(Intercept)	0.000	0.000	0.000
Log (Median home value)	0.158	0.105	− 0.167
Log (Vacancy rate)	0.044	0.057	0.090
**Low-wage jobs-housing fit**	0.170		
**Medium-wage jobs-housing fit**		0.171	
**Jobs-housing ratio**			0.066
Employment center dummy	0.553	0.697	0.463
Log (Distance from city hall)	0.284	0.350	0.173
Employment center* Log (Distance from city hall)	− 0.415	− 0.621	− 0.425
Urban neighborhood	− 0.062	− 0.025	− 0.047
Within 0.5 miles of rail station	0.073	0.040	0.018
Ratio of low-wage and medium-wage jobs relative to high-wage jobs	− 0.137	− 0.098	− 0.061
Competition for low-wage jobs	0.119		
Competition for medium-wage jobs		0.082	
Competition for high-wage jobs			0.073

**Table 4:** Estimation results for OLS models for Lower-Wage and Medium-Wage Workers’ Commute Distance with Jobs-Housing Ratio as the Variable of Interest

	Lower-wage workers		Medium-wage workers	
	OLS	Std. Error	OLS	Std. Error
Intercept	**− 1.443**	**0.128**	-0.617	**0.102**
***Housing Characteristics***				
Log (Median home value)	**0.207**	**0.019**	**0.107**	**0.015**
Log (Vacancy rate)	**0.031**	**0.012**	**0.029**	**0.010**
Jobs-housing ratio	**0.045**	**0.006**	**0.019**	**0.005**
***Locational Characteristics***				
Employment center dummy	**0.317**	**0.155**	**0.377**	**0.123**
Log (Distance from city hall)	**0.240**	**0.014**	**0.211**	**0.012**
Employment center*Log (Distance from city hall)	− 0.049	0.033	**-0.072**	**0.026**
Urban neighborhood	**− 0.034**	**0.008**	**-0.018**	**0.007**
Within 0.5 miles of rail station	**0.051**	**0.013**	**0.021**	**0.010**
***Employment Characteristics***				
Ratio of low-wage and medium-wage jobs relative to high-wage jobs	**− 0.006**	**0.001**	**-0.004**	**0.001**
Competition for low-wage jobs	**0.010**	**0.002**		
Competition for medium-wage jobs			**0.006**	**0.001**
R^2^	0.275		0.228	

Values shown in bold are significant at *p* < 0.05

## Footnotes

1 Workers in Los Angeles have a shorter average commute distance and better jobs-housing balance than workers in other more sprawling metropolitan areas (Schleith et al. 2019).

2 Seven out of 2,929 census tracts (0.24% of the census tracts) in the Los Angeles MSA did not have any employment reported in the LODES dataset and were excluded from this analysis.

3 Microdata from the 2015–2019 5-Year American Community Survey show that among employed workers 16 years and older, 24 percent have wages below $1,250, 30 percent have wages between $1,250 and $3,333, and 46 percent of wages greater than $3,333 (Ruggles et al. 2021).

4 There is no standard definition of the geographic area suitable for measuring jobs-housing balance. At some spatial scale all housing and workers are in perfect balance. Benner and Karner (2016) use the 0.5 and 3-mile buffers based on walking and biking as the primary means of travel to work, drawing from both national and California data to support the buffers. Few workers in the Los Angeles MSA bike or walk to work (3.3%) (Ruggles et al. 2021); however, the ability to walk or bike to work can serve as one measure of a local jobs-housing shed. Studies also show that statistical models based on an impedance factor are not particularly sensitive to the specific value (Blumenberg and Ong 1998; Bunel and Tovar 2014).

5 For a detailed discussion of the differences between the LODES data and data from American Community Survey commuting products, see Graham et al. (2014).

6 This finding is drawn from the standardized coefficients of the OLS models (Table 1 in the appendix).

7 The dummy variables for location in an employment center, an urban neighborhood, and within 0.5 miles of rail station are held at one for the estimation of the effect of jobs-housing fit on commute distance in Fig. 4.
